# Trends for Surgical Treatment of Testicular Varicocele: A German Whole-population Analysis of Inpatient Procedures from 2006 to 2021

**DOI:** 10.1016/j.euros.2025.03.001

**Published:** 2025-03-28

**Authors:** Cem Aksoy, Philipp Reimold, Philipp Karschuck, Subhajit Mandal, Nicole Eisenmenger, Christer Groeben, Aristeidis Zacharis, Johannes Huber, Luka Flegar

**Affiliations:** a Department of Urology, Philipps-University Marburg, Marburg, Germany; b Reimbursement Institute, Hürth, Germany

**Keywords:** Varicocele, Health care, Laparoscopic, sexual health

## Abstract

**Background and objective:**

Testicular varicocele (TVC) is a common benign finding in men of all ages. Indications for TVC repair are abnormal sperm parameters and testicular pain. The optimal surgical method for TVC repair is still a matter of controversy. The aim of our study was to analyze the current situation and trends for TVC surgery in Germany.

**Methods:**

The German reports for hospital quality were analyzed from 2006 to 2021. Linear regression models were applied to detect trends over time. Outpatient procedure rates were estimated.

**Key findings and limitations:**

A total of 38 653 inpatient TVC surgeries were included. Total varicocelectomy procedures decreased from 3456 in 2006 to 2165 in 2021 (−37.4%; *p* < 0.001). The proportion of open TVC procedures remained constant at ∼25%. The proportion of laparoscopic TVC procedures increased from 25% in 2006 to 45% in 2021 (*p* < 0.001), while the sclerotherapy rate for TVC treatment decreased from 47% in 2006 to 29% in 2021 (*p* < 0.001). In 2006, 543 hospitals offered TVC treatment, which decreased to 466 hospitals in 2021 (−14.2%). Among patients undergoing surgical TVC procedures in 2021, 75% were younger than 35 yr. We estimated an increase in outpatient procedures from 365 cases in 2013 to 1330 cases in 2021 (*p* < 0.001). The estimation for outpatient procedures represents a study limitation.

**Conclusions and clinical implications:**

Laparoscopic TVC treatment is the dominant surgical approach. However, one in three patients receives sclerotherapy for TVC in Germany. While fewer hospitals offer the treatment, we observed a trend for centralization, with an increase in high-caseload departments, as well as an increasing trend for outpatient treatment.

**Patient summary:**

TVC is a collection of swollen veins in the testicle. We analyzed treatment trends in Germany for this condition from 2006 to 2021. There was an increase in the use of keyhole surgery over time, and we estimate that the percentage of day-case surgeries also increased.

## Take Home Message

Our analysis of caseload trends for testicular varicocele treatment in Germany between 2006 and 2021 shows that minimally invasive laparoscopic surgery is now the most common inpatient procedure. Some 75% of patients were younger than 35 yr at the time of treatment.

## 1 Introduction

Conditions affecting the scrotum can have different causes and present with various symptoms. The clinical presentation varies from an unpleasant sensation at rest to pain of varying intensity on exertion. Causes range from trauma and infection to benign or malignant scrotal masses. An important differential diagnosis among possible causes is testicular varicocele (TVC). The underlying pathophysiology is characterized by abnormal dilatation of the pampiniform venous plexus in the scrotum due to a pathological venous reflux in the internal spermatic vein.

TVC is a common condition that is frequently observed in young men and adults. The TVC prevalence is approximately 15% in adults and 14% for adolescents aged 15–19 yr [1]. An analysis of TVC incidence revealed that the trend has hardly changed over the past few decades [2]. Causes of TVC can be divided into primary and secondary categories. Primary TVC is often caused by left-sided anatomic variations such as the angle of confluence between the testicular vein and the renal vein. Secondary TVC usually occurs because of a retroperitoneal mass hindering venous outflow of the testis [3]. Regardless of the clinical presentation, important complications worth mentioning are infertility and altered hormonal status in affected individuals. Among men with primary infertile, the incidence of TVC is estimated as 35–44% [4]. TVC severity is graded on a clinical scale that ranges from subclinical (stage 0) to visible varicocele without Valsalva (stage III). An increase in TVC grade has a negative effect on testicular function in men. Hormonally, this affects follicle-stimulating hormone, inhibin B, and luteinizing hormone [5]. According to a retrospective study by Hsiao et al [6], testosterone levels are also affected in infertile and/or hypogonadal men with TVC. Microsurgical repair leads to a significant increase in serum testosterone levels, independent of TVC grade [6].

Surgical treatments for TVC are associated with improvements in sperm quality, decreases in DNA damage, and improvements in hormonal status, independent of possible clinical improvements [7,8]. Surgical treatment options include laparoscopy, open surgery or microsurgery, and sclerotherapy, which differ in their approaches and invasiveness [9]. In particular, sclerotherapy and inguinal open surgery are procedures that can be routinely performed on an outpatient basis [10,11].

The aim of our study was to analyze surgical trends for inpatient TVC treatment in Germany from 2006 to 2021. Owing to the multiple treatment options for TVC and the constant evolution of medical standards in terms of modernization, insurance coverage, and health care trends, the study covers all inpatient TVC treatments in Germany.

## 2 Patients and methods

The German quality reports for hospitals were analyzed for the study. The methodology for extracting data and identifying cohorts has been described in detail in previous studies [9].

### 2.1 Quality reports

Since 2005, German hospitals have been required by law to provide details about their work via quality reports. We used the reimbursement.INFO analysis tool (RI Innovation GmbH, Hürth, Germany) to extract billing data in the form of diagnosis-related groups from DESTATIS (Statistisches Bundesamt) on hospitals performing TVC surgeries between 2006 and 2021. For the present analysis, we used OPS (German adaption of the International Classification of Procedures in Medicine) codes 5-630.0 (scrotal sclerotherapy of varicocele spermatica), 5-630.1 (open inguinal resection of varicocele spermatica), 5-630.2 (open lumbar resection of varicocele spermatica), 5-630.3 (open abdominal resection of varicocele spermatica), and 5-630.4 (laparoscopic resection of varicocele spermatica). Codes 5-630.1, 5-630.2, and 5-630.3 were grouped together for analysis as an open surgical approach. Maps were generated using EasyMap 11.1 standard edition software (Lutum+Tappert DV-Beratung GmbH, Bonn, Germany).

### 2.2 Estimation of outpatient procedures

The DESTATIS database only provides information on inpatient treatments. Therefore, we used the year with the highest TVC procedure frequency (2008) as a reference for the total number of TVCs treated. In relation to this reference, we estimated the proportion of outpatient TVC treatments for subsequent years, assuming that the number of cases remained constant, as reported in literature [2].

### 2.3 Statistical analysis

Data are presented as absolute and relative frequencies. Linear regression models were used to identify trends over time. Statistical significance was defined as *p* < 0.05. The statistical analysis was performed with SPSS version 28 (IBM Corp., Armonk, NY, USA).

### 2.4 Ethics considerations

The data presented in this study were collected in compliance with the Declaration of Helsinki. Since the data extracted from the DESTATIS database were appropriately anonymized and deidentified before being released, no informed consent from patients was necessary, and no additional ethics statement was required for the study.

## 3 Results

We included 38 653 inpatient TVC treatments in our analysis. Three out of four patients undergoing a surgical TVC procedure in 2021 were younger than 35 yr (Supplementary Fig. 1).

**Fig. 1:**
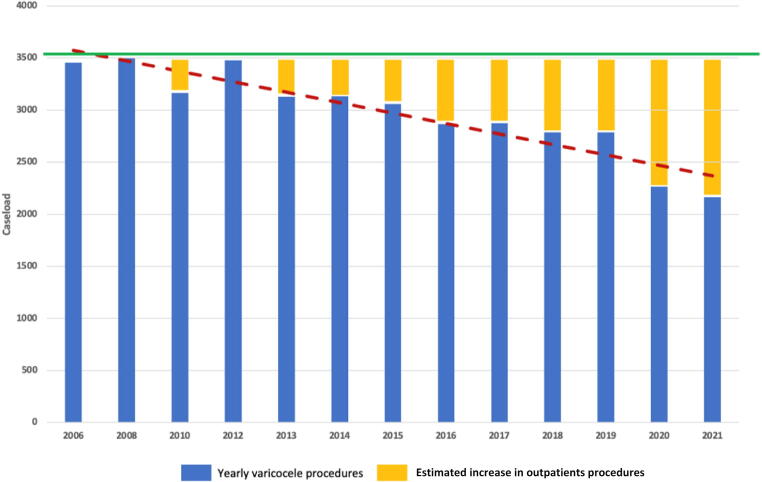
Caseload for varicocelectomy between 2006 and 2021. The green line shows the most frequent number of inpatient testicular varicocele (TVC) procedures in 2008 as an estimated reference for the total number of TVCs treated. The red dashed line represents the trend for inpatient TVC treatment during the study period, with an annual decrease. The yellow bars represent the estimated increase in outpatient procedures.

**Fig. 2:**
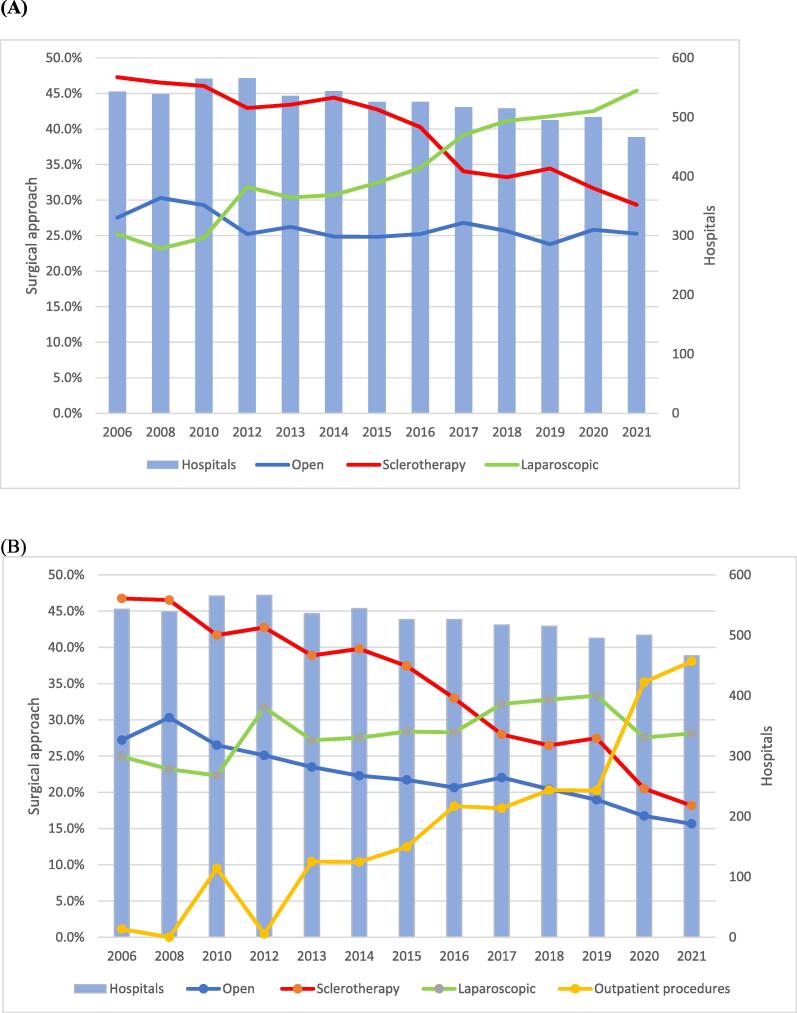
(A) Surgical approach for inpatient testicular varicocele (TVC) treatment and hospitals offering the therapy between 2006 and 2021. (B) Surgical approach for inpatient TVC treatment, proportion of outpatient procedures, and hospitals offering TVC therapy between 2006 and 2021.

**Fig. 3:**
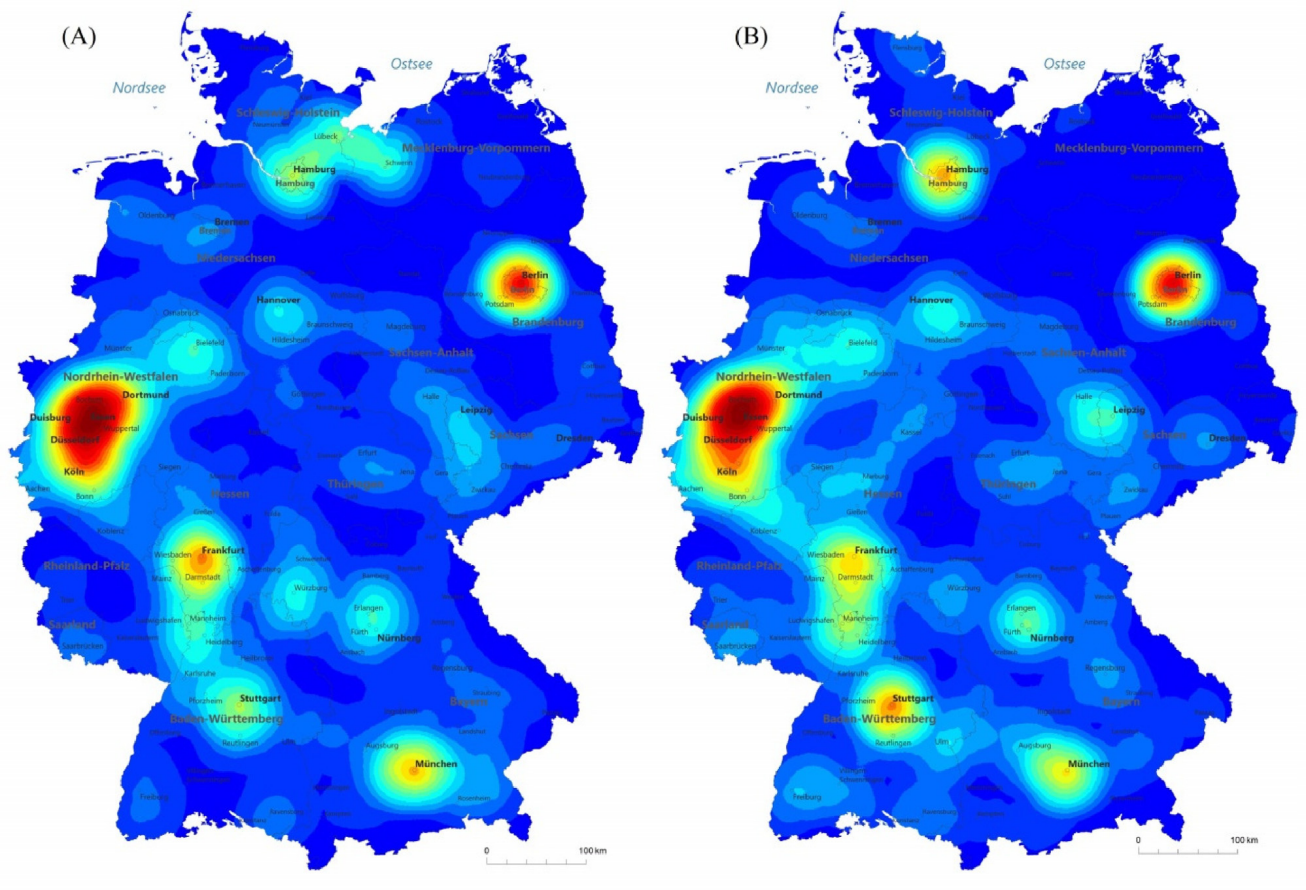
Heatmaps representing all inpatient surgical therapies for testicular varicocele in (A) 2006 and (B) 2021. Red indicates regions with hospitals with a high caseload, while blue indicates regions with hospitals with a low caseload.

**Fig. 4:**
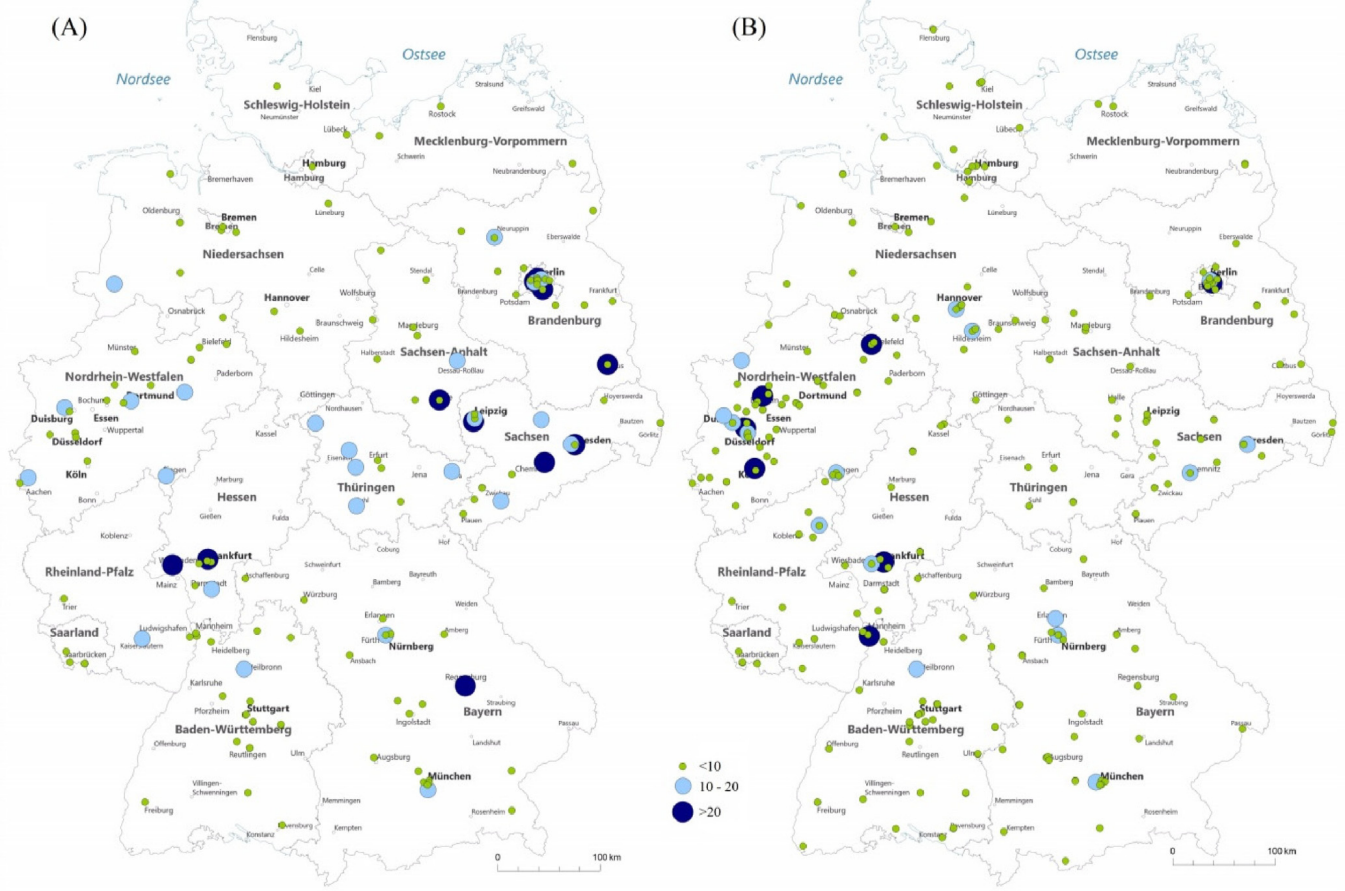
Geographic distribution of hospitals performing laparoscopic surgery for testicular varicocele in (A) 2006 and (B) 2021.

Total inpatient varicocelectomy procedures decreased from 3456 in 2006 to 2165 in 2021 (−37.4%; *p* < 0.001; Fig. 1). We estimated an increase in outpatient TVC procedures from 365 cases in 2013 to 1330 cases in 2021 (*p* < 0.001). The proportion of open TVC procedures decreased slightly to ∼25% in 2021 (*p* = 0.01). The proportion of laparoscopic TVC surgeries increased from 25% in 2006 to 45% in 2021 (*p* < 0.001), while the proportion of sclerotherapy treatments for TVC decreased from 47% in 2006 to 29% in 2021 (*p* < 0.001; Fig. 2A). Fig. 2B shows that the estimated proportion of outpatient procedures increased to 38% in 2021 (*p* < 0.001), while the proportion of laparoscopic TVC surgeries increased from 25% in 2006 to 28% in 2021 (*p* < 0.001). Use of the open inguinal approach increased from 34% in 2006 to 79% in 2021(*p* < 0.001), while use of the open abdominal approach decreased from 56% in 2006 to 17% in 2021 (*p* < 0.001). Use of the open lumbar approach decreased from 9% in 2006 to 4% in 2021 (*p* < 0.001; Supplementary Fig. 2). TVC treatment was offered by 543 hospitals in 2006, which decreased to 466 hospitals in 2021 (−14.2%). The heatmaps in Fig. 3 for 2006 and 2021 show an increase in high-caseload centers in Germany over the study period. Laparoscopic TVC treatment was offered all over Germany. The hospital distribution by laparoscopic TVC case volume was 124 hospitals (77%) with <10 cases, 24 hospitals (15%) with 10–20 cases, and 12 hospitals (8%) with >20 cases in 2006, and 230 hospitals (91%) with <10 cases, 16 hospitals (6%) with 10–20 cases, and seven hospitals (3%) with >20 cases in 2021 (Fig. 4).

## 4 Discussion

We investigated trends for the surgical treatment of TVC in Germany from 2006 to 2021. Our results show that the laparoscopic approach is now the most common inpatient procedure and that 75% of patients treated for TVC are younger than 35 yr. One in three patients received sclerotherapy for TVC repair. Furthermore, a trend for centralization of TVC surgery was noted in recent years.

While varicocelectomy is by far the most common surgical treatment for male infertility, the most suitable surgical approach for TVC repair remains a subject of debate. The main goals of any technique are to maintain or enhance testicular function, to eliminate TVC with a low recurrence rate, to minimize intraoperative and postoperative complications and morbidity, and to achieve a cost-effective solution [12]. Various techniques are available for TVC treatment, including open surgical ligation of the spermatic vein, retrograde or anterograde sclerotherapy, microsurgery, and laparoscopy [13]. Each of these methods comes with a unique set of benefits and drawbacks. The surgical approach chosen depends on the experience of the clinic and on patient comorbidity and previous surgeries. Outcomes in terms of a low recurrence rate are best for microscopic subinguinal treatment [14]. A comparison of laparoscopic and microsurgical treatments revealed lower complication rates and an increase in sperm concentration with microsurgical treatment [15]. A benefit of sclerotherapy is that it can be performed under local anesthesia.

Our analysis showed that the share of laparoscopic TVC treatment increased significantly from 25% in 2006 to 45% in 2021 and that it is the dominant surgical approach. In the early 1990s, laparoscopic transperitoneal Palomo varicocelectomy was introduced and has since gained broad recognition as a secure, uncomplicated, and minimally invasive procedure for both adults and children.

Hassan et al [16] reported that hydrocele occurrence was the most common postoperative complication after laparoscopic surgery, occurring in up to 25% of patients. In cases involving obese patients, the laparoscopic transperitoneal approach is especially advantageous as it allows excellent visualization of the spermatic vessels [12]. Jukic et al [17] reported that the rate of TVC recurrence was zero after laparoscopic treatment and 4.1% after open treatment in their single-center study. Several studies have described advantages of laparoscopic TVC treatment in terms of shorter hospital stays and shorter operation times [17,18]. While our study indicates that laparoscopic varicocelectomy has been the primary approach in Germany in recent years, a population-level analysis of trends in the diagnosis and management of TVC in the USA revealed that open varicocelectomy continued to be the predominant approach for TVC repair in 2019 [19]. The authors speculated that this trend could be related to several physician-level issues, as surgeons may not be adequately trained in laparoscopy and microsurgery. Park et al [20] reported similar results for repeat varicocelectomy in 2021, with the open inguinal approach most frequently used. Laparoscopic surgery is associated with a high degree of difficulty and a long learning curve, and is usually difficult for kidney and prostate procedures [21]. By contrast, laparoscopic surgery for TVC represents a manageable urological operation for novice surgeons. Owing to the rapid development of robot-assisted surgery, laparoscopic procedures are increasingly being replaced by robot-assisted procedures in urology [22]. This development is a great pity, as laparoscopic treatment of TVC is a procedure that is very attractive in terms of urologist training and in the context of inpatient treatment. By contrast, robotic repair of TVC is associated with high costs and economic inefficiency [23]. The high proportion of laparoscopic treatments in Germany may be partly explained by decreasing costs [24].

Our results show that one in three patients received sclerotherapy for TVC repair. A study from 2019 compared conventional sperm parameters after surgical varicocelectomy versus sclerotherapy [25]. Sperm concentrations significantly improved with both approaches, but there was a statistically significant increase in progressive and total sperm motility only for patients undergoing sclerotherapy. Moreover, the incidence of TVC recurrence was notably lower following sclerotherapy in comparison to surgical varicocelectomy [25]. Jargiello et al [26] also showed that sclerotherapy may be superior to surgery in the treatment of postsurgical TVC because of its ability to detect gonadal vein variants. Our results showed that the proportion of open TVC procedures remained constant at ∼25% during the study period. Conventional open varicocelectomy can be performed at various anatomic levels [27]. In the present study, inguinal varicocelectomy was the most common open approach. Alternatively, TVC can be repaired via retroperitoneal high ligation of the testicular vein [27]. Complications associated with open TVC repair range from mild hematoma and wound infection to hydroceles and testicular atrophy [27]. Another option for surgical TVC repair is inguinal or subinguinal microsurgical varicocelectomy. Advantages of the microsurgical approach include very low recurrence and persistence rates [27,28].

Our analysis showed a decreasing trend for varicocelectomy, from 3456 procedures performed in 2006 to 2165 in 2021. We postulate that this trend is related to a shift in performing varicocelectomy on an outpatient basis in recent years. Therefore, we estimated the proportion of outpatient procedures for the study period and noted a significant increase from 2006 to 2021. We also observed a significant decrease in TVC treatment during the COVID-19 pandemic. While 2265 TVC treatments were performed in 2020, 2165 were performed in 2021. A similar trend for fewer surgical procedures during the COVID-19 pandemic has been observed in several studies on elective procedures [29,30].

### 4.1 Current and future trends

The growing availability of robotic surgical systems has led to expansion of their use in performing varicocelectomy procedures in recent years. The first robot-assisted varicocelectomy with the da Vinci system was performed by Corcione et al in 2005, as reviewed by Marte [31]. In 2009, the first robot-assisted laparoscopic varicocelectomy in a pediatric patient was reported. The authors stated that the procedure was technically feasible with no intraoperative complications, but the cost effectiveness in comparison to laparoscopic varicocelectomy has to be investigated in the future [32]. Darves-Bornoz and colleagues [33] described the practical advantages of robot-assisted varicocelectomy over microsurgery for male infertility, including a reduction in or elimination of tremor, and three-dimensional visualization. In Germany, robotic varicocelectomy is not economically sound. Moreover, laparoscopic varicocelectomy represents the ideal approach for teaching the technique.

The shift towards centralization of TVC care has many consequences that can be illustrated by clinical and logistic examples. Although centralization of TBC treatment can achieve a higher quality of care, it can also limit access to treatment, as patients have to travel further for treatment and follow-up, which represents a financial and a time burden.

### 4.2 Limitations

Several study limitations have to be acknowledged. The quality reports for German hospitals lack clinical information such as patient characteristics. Initially they were released every 2 yr, so the years 2007, 2009, and 2011 are missing. Furthermore, only inpatient treatments were analyzed, and the suggested shift in treatments to outpatient procedures cannot be directly extracted from our data set. Consequently, we had to estimate the proportion of outpatient treatments. This estimate comes with inaccuracy in terms of numbers and we cannot specify the actual number of outpatient procedures performed. Estimation of outpatient TVC treatments may lead to over- or underestimation of outpatient cases in the time period examined, with potential for different results for treatment trends that could result in different study conclusions. In addition, the quality reports may be subject to documentation errors as they are prepared by the hospitals during routine care. Unfortunately, there is no OPS code available for microsurgical TVC repair. However, we speculate that this is represented among the open procedures. Finally, the OPS code for sclerotherapy does not differentiate between antegrade and retrograde approaches. However, the present population-based study is the first to investigate inpatient surgical approaches for TVC over an extensive time span of 16 yr.

## 5 Conclusions

Our study revealed that laparoscopic procedures have been the dominant surgical approach for inpatient TVC treatment in Germany in recent years. One in three patients received sclerotherapy for TVC. We also observed a centralization trend, with an increase in high-caseload departments, as well as an increasing trend for outpatient TVC treatment.

  ***Author contributions***: Cem Aksoy had full access to all the data in the study and takes responsibility for the integrity of the data and the accuracy of the data analysis.

  *Study concept and design*: Aksoy.

*Acquisition of data*: Eisenmenger.

*Analysis and interpretation of data*: Groeben.

*Drafting of the manuscript*: Aksoy.

*Critical revision of the manuscript for important intellectual content*: Huber.

*Statistical analysis*: Flegar.

*Obtaining funding*: None.

*Administrative, technical, or material support*: Karschuck.

*Supervision*: Reimold.

*Other* (*proofreading*): Mandal, Zacharis.

  ***Financial disclosures:*** Cem Aksoy certifies that all conflicts of interest, including specific financial interests and relationships and affiliations relevant to the subject matter or materials discussed in the manuscript (eg, employment/affiliation, grants or funding, consultancies, honoraria, stock ownership or options, expert testimony, royalties, or patents filed, received, or pending), are the following: Cem Aksoy is founder and a member of PATE e.V. (Prevention and Advocacy of Testicular Education, nonprofit association) and reports grants from Merck and Apoghepha. Nicole Eisenmenger is founder and director of RI Innovation GmbH. Johannes Huber reports grants and non-financial support from Intuitive Surgical, Takeda, Janssen, Apogepha, and Coloplast outside the submitted work, and a role as a medical board member for the Urological Foundation for Health. Luka Flegar is a consultant for BK Medical and reports grants from Novartis and Astellas. The remaining authors have nothing to disclose.

  ***Funding/Support and role of the sponsor*:** None.

  ***Ethics considerations:*** This study was conducted in accordance with the latest version of the Declaration of Helsinki. Ethics approval for the study was not required in accordance with local and national guidelines. For data protection reasons, diagnostic (ICD) data or intervention number (OPS) data with a number of ≤3 in the quality reports do not indicate the actual number, but the number 1. All data used are anonymized, so no further ethics committee approval was required. Written informed consent was not needed.

  ***Data sharing statement***: The German hospital quality reports are publicly accessible. The data sets generated for and/or analyzed during the current study are available from the corresponding author on reasonable request.
