# Supplementary material for: Trends for Surgical Treatment of Testicular Varicocele: A German Whole-population Analysis of Inpatient Procedures from 2006 to 2021
Source: Eur Urol Open Sci. 2025 Mar 28;75:29–36. doi: 10.1016/j.euros.2025.03.001 (PMC11992527; doi:10.1016/j.euros.2025.03.001)
Supplement: Supplementary Data 1 [file mmc1.docx]

**Supplementary Fig. 1** Age distribution in 2006 and 2021 for all varicocele procedures.

**Supplementary Fig.2.** Share of open ingunial, abdominal, and lumbal approaches.
